# Low‐dose total skin electron beam therapy in erythrodermic mycosis fungoides and Sézary syndrome: Results From the Prospective S‐MISR Study

**DOI:** 10.1111/ddg.15851

**Published:** 2025-08-23

**Authors:** Khaled Elsayad, Christian Kandler, Jan Jakob Siats, Niklas Pepper, Moritz Fabian Danzer, Gabor Dobos, Elisabeth Livingstone, Susanne Melchers, Johannes Kleemann, Julia Hyun, Claudia Pföhler, Peter von den Driesch, Jan P. Nicolay, Rudolf Stadler, Hans Theodor Eich

**Affiliations:** ^1^ Department of Radiation Oncology University Hospital Münster Münster Germany; ^2^ Institute of Biostatistics and Clinical Research University of Münster Münster Germany; ^3^ Clinic for Dermatology Venereology and Allergology Charité – Universitätsmedizin Berlin corporate member of Freie Universität Berlin and Humboldt Universität zu Berlin Berlin Germany; ^4^ Department of Dermatology University Hospital Essen Essen Germany; ^5^ Department of Dermatology Venereology and Allergology University Medical Center Mannheim University of Heidelberg Mannheim Germany; ^6^ Departments of Dermatology Venereology and Allergy Goethe University Frankfurt am Main Germany; ^7^ Department of Dermatology Venereology and Allergology Helios St. Johannes Hospital Duisburg Duisburg Germany; ^8^ Saarland University Medical Center Deparment of Dermatology Homburg/Saar Germany; ^9^ Department of Dermatology Klinikum Stuttgart Bad Cannstatt Stuttgart Germany; ^10^ University Department for Dermatology Venerology Allergology and Phlebology Skin Cancer Center Johannes Wesling Medical Center UKRUB University of Bochum Minden Germany

**Keywords:** blood involvement, health‐related quality of life, immunotherapy, leukemic type, pruritus, Radiotherapy, S‐MISR registry

## Abstract

**Background:**

Erythrodermic mycosis fungoides (eMF) and Sézary syndrome (SS) often show delayed and non‐durable clinical responses to systemic therapies.

**Patients and Methods:**

A total of 35 patients with eMF or SS were treated with total skin electron beam therapy (TSEBT). Response rates, patient‐reported outcomes, survival data, median time to next treatment (TTNT), and progression‐free survival (PFS) were evaluated.

**Results:**

The analysis included 21 patients with SS and 14 with eMF. The median radiation dose was 12 Gy. At 3 months post‐TSEBT, the overall response rate was 89%. A total of 25 patients (71%) required subsequent systemic therapy. The median TTNT was 20 months, and the median PFS was 14 months. Patients reported marked reductions in pruritus, decreased skin disease burden, and improved health‐related quality of life scores. Notably, both TTNT and PFS were longer in patients who received subsequent therapy compared to those who did not. Grade ≥3 radiation‐related toxicity was observed in 6% of patients. In the translational arm of the study, several potential peripheral blood biomarkers were identified.

**Conclusions:**

Low‐dose TSEBT is an effective treatment option for patients with eMF and SS, facilitating subsequent therapies and enabling long‐term disease control. The treatment is associated with significant quality‐of‐life improvements and low toxicity.

## INTRODUCTION

Sézary syndrome (SS) is a rare primary cutaneous T‐cell lymphoma, with an annual incidence of less than 0.2 cases per million people.^1^, ^2^ In addition to erythroderma, SS is characterized by circulating malignant T cells that mirror the skin infiltrate, as well as evidence of an increased blood tumor burden (B2 stage) with aberrant T‐cell phenotypes, such as CD4^+^/CD26^−^ or CD4^+^/CD7^−^ populations, identified by flow cytometry.^3^ A small proportion of patients with mycosis fungoides (MF) (< 5%) present with diffuse erythroderma (T4 stage) but a low blood tumor burden (B0–B1 stage).^4^ The prognosis of SS is poorer than that of patients with MF.^5^ The prognostic significance of erythrodermic MF (eMF), however, remains unclear.^3^ Clinically, patients typically present with moderate to severe skin involvement and a marked deterioration in quality of life (QoL).^6^ Most SS patients are diagnosed *de novo*; however, a small proportion of cases might emerge secondary to MF.^7^ Treatments for patients with eMF and SS differ across centers and usually provide delayed and non‐lasting clinical benefits.^7^ For the treatment of eMF recommendations for SS may apply.^8^ Additionally, radiotherapy (RT) might be associated with improvement of clinical symptoms.^9^ Limited low‐dose total skin electron beam therapy (TSEBT) trials included eMF and SS patients with reasonable results.^10^ Moreover, TSEBT might reduce tumor burden in the peripheral blood.^11^, ^12^ Few retrospective studies suggest that soluble biomarkers might correlate with disease severity,^13^ and may reflect disease activity.^14^, ^15^

In this analysis, we aim to explore the efficacy of a low‐dose TSEBT regimen and the impact of subsequent treatment in patients with eMF and SS. In addition, we describe the exploratory analysis of potential biomarkers from baseline to the follow‐up.

## PATIENTS AND METHODS

### Study design

**FIGURE 1:**
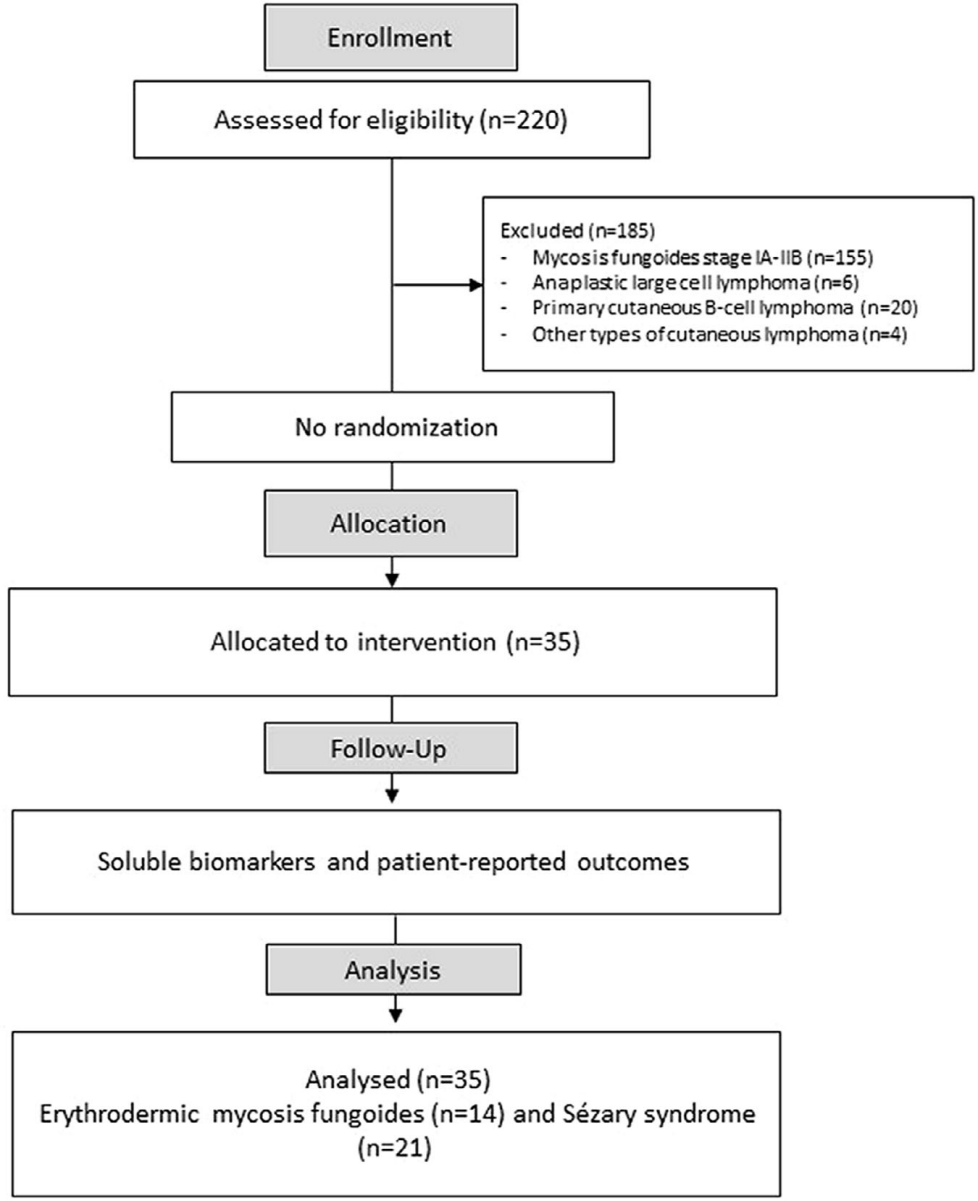
CONSORT flowchart for erythrodermic mycosis fungoides and Sézary syndrome patients received low‐dose total skin electron beam therapy (n = 35).

This study is a prospective observational study of patients with eMF and SS. Patients referred from affiliated dermatological centers in Germany were enrolled (TrialSearch (WHO) and the German Clinical Trials Register Number: DRKS00030375). Patients with clinically and histologically confirmed active eMF (stage IIIA–IIIB) and SS (stage IVA) were included in this analysis (Figure 1).

### Patients

The inclusion criteria were a histologically confirmed diagnosis of eMF and SS, inadequate response to at least one previous therapy, age ≥ 18 years, washout duration of 4 weeks after systemic treatment and 2 weeks after topical treatment, life expectancy of more than 6 months, and inadequate response to at least one previous therapy. The exclusion criteria included a history of or active skin malignancy, active or prior non‐infectious pneumonitis requiring steroid treatment, pulmonary fibrosis, pregnancy or lactation, and patients awaiting stem cell transplantation. In this observational study, the subsequent therapies with the intent to maintain remission were administered within 3 months following TSEBT after reaching a clinical response. The subsequent treatment was continued until there is a progression or unacceptable toxicity.^16^ The administration of a pre‐planned subsequent treatment was not considered as a TTNT event.^17^

From September 2019 to August 2024, a total of 220 patients with primary cutaneous lymphoma were recruited from the S‐MISR registry across nine treatment centers, all of whom had provided informed consent. In this analysis, 21 treated patients had SS, including ten with de novo SS and eleven with MF that had progressed to SS. Additionally, 14 patients were diagnosed with eMF. Immunohistochemistry CD30 expression was available in 27 patients, with positive results in nine patients (33%) with a median expression of 10% (IQR 5–30). All patients had active or progressive disease following prior treatments (median of 3 treatments, range of 1–8). The radiation dose was determined by the treating radiation oncologist based on individual clinical circumstances. No patients in our cohort received concomitant therapy. The median radiation dose was 12 Gy (range, 8 to 12 Gy), administered using a six‐dual‐field technique. Due to the COVID‐19 pandemic, 14 patients (40%) received ultra‐hypofractionated treatment regimens.^18^ Thirty‐one (89%) patients received supplementary electron fields to areas shielded during TSEBT. At the same time, 19 patients (54%) received local boost radiotherapy to pathologically enlarged axillary and inguinal lymph nodes, with a median dose of 9 Gy (range, 4–30 Gy). Baseline assessments included medical history and QoL questionnaires. TSEBT toxicities and QoL were assessed weekly during radiation, after 6–8 weeks, after 12 weeks, then every 3 months until relapse or progression. A total of 29 patients (83%) completed the Skindex‐29 and EORTC QLQ‐C30 (Version 3.0) questionnaires before and within 12 weeks following TSEBT.

Additionally, blood samples from all patients have been acquired at baseline and follow‐up for exploratory blood tests. Peripheral blood samples were sent for laboratory testing within 4 hours of collection. Analyses included routine blood parameters, peripheral blood cell counts, β_2_‐microglobulin, lactate dehydrogenase (LDH), C‐reactive protein (CRP), soluble interleukin (IL)‐2 receptor (sIL‐2R), soluble interleukin‐6 (sIL‐6), neopterin, tumor necrosis factor (TNF)‐α, and immunoglobulin concentrations. Additional blood samples (7 mL) were collected in serum tubes before and after radiotherapy. Samples were centrifuged at 2500  ×  g for 10 min. The serum fractions were aliquoted into cryovials (Greiner, Kremsmünster, Austria) and stored at –80°C before additional analysis of soluble programmed cell death ligand‐1 (sPD‐L1), soluble CD30 (sCD30), soluble thymus and activation‐regulated chemokine (sTARC) using enzyme‐linked immunosorbent assay (ELISA) kits (Bio‐Techne, Wiesbaden, Germany)^38^.

### Outcomes

International response criteria have been used for response evaluation.^19^ Responders at 3 months included patients with complete response (CR), very good partial remission (VGPR), and partial remission (PR), while non‐responders included patients with stable disease (SD) or progressive disease (PD). The primary endpoints were response rates, in accordance with international recommendations.^3^, ^17^, ^19^ Secondary outcomes were time to next treatment (TTNT) and progression‐free survival (PFS; the period from TSEBT initiation to progression, relapse, or death). Exploratory objectives included quality of life assessment and changes in blood parameters from samples collected at baseline and follow‐up, aiming to evaluate shifts in potential biomarker levels.

### Statistical Analysis

We conducted statistical analyses using IBM SPSS Statistics version 29.0. The responses are presented as both absolute numbers and relative values. We used Chi‐square tests or Fisher's exact tests to examine associations between categorical variables. Continuous variables are described by their medians, interquartile ranges (IQR), and quartiles. Kaplan–Meier curves were used to estimate median PFS and TTNT, with standard methods applied to calculate the corresponding confidence intervals (CIs).^20^ The log‐rank test was employed to compare time‐to‐event endpoints between different groups. The hazard ratio from the Cox proportional hazards model and its corresponding CIs are an accompanying measure of effect size. Differences in biomarkers before and after treatments were reported using median values, with p‐values calculated using the Wilcoxon signed‐rank test. The correlation between baseline values and clinical parameters was assessed using Spearman rank correlation. A Bonferroni correction was applied to compare mSWAT and Skindex‐29 (sub)scores before and after TSEBT. Corrected p‐values < 0.05 were considered statistically significant.

## RESULTS

Patient demographics as well as clinical and biochemical characteristics are summarized in Table 1. The median age of the whole cohort was 68 years (IQR 60–76). The median follow‐up duration was 9 months (IQR 4–24). Patients had a substantial mSWAT improvement within 6 weeks of TSEBT initiation (p < 0.001). The median mSWAT score before radiation was 99 (IQR 80–100) versus 20 (IQR 6–41) in the sixth week and 12 (IQR 5–29) in third month after TSEBT. Regarding the lymph nodes, the response rate was 89% in the entire cohort, with a 100% response rate in patients who received local RT to the lymph nodes compared to patients who did not receive nodal irradiation (57%, p = 0.013). Regarding blood involvement, only 20 patients had paired blood tests, with a response rate of 80% (40% CR of aberrant T‐cell populations after TSEBT and 40% had partial response), while 20% had stable aberrant T‐cell populations after TSEBT.

The overall response rate (ORR) at 3 months was 89%, with 14 PR (40%), 15 VGPR (43%), and two CR (6%). There was no meaningful difference in ORR between eMF and SS (93% vs. 86%, respectively). In comparison, the rate of SD was 7% in the eMF group vs. 9% in the SS group. Only one patient in the SS cohort progressed directly after TSEBT. Twenty‐five (71%) patients received subsequent treatment after TSEBT. Examples of clinical responses can be seen in the online supplementary Figures S1 and S2.

In the entire cohort, the median TTNT was 20 months (95% confidence interval [CI]: 10–30), and the median PFS was 14 months (95% CI: 4–24). No significant differences in response rates, TTNT, or PFS were observed with respect to radiation dose or fractionation schedule. Seven patients received a second course of TSEBT after a median interval of 13 months. Following radiotherapy, the 5‐year overall survival was 92% in patients with eMF and 36% in those with SS.

### Subsequent systemic treatments

A total of 31 responders were evaluated for the benefit of subsequent therapy. The most common subsequent therapy was mogamulizumab therapy (n = 9), followed by oral retinoids (n = 5), methotrexate therapy (n = 5), pegylated interferons alpha (n = 3), extracorporeal photopheresis (n = 2), and others (n = 7). The median follow‐up period was similar between the subsequent therapy group and the group without subsequent therapy. For patients receiving subsequent therapy, the median TTNT was 30 months (95% CI: 14–46, p = 0.001). In contrast, responders without subsequent therapy had a median TTNT of only 6 months (95% CI: 2–10). Additionally, the median PFS for patients in the subsequent therapy group was 23 months (95% CI: 9–37), while it was only 5 months (95% CI: 2–8; p < 0.001) for those without subsequent therapy. This favorable outcome was also observed in subgroup analysis for the eMF cohort (42 vs. 13 months, p = 0.036) and SS cohort (20 vs. 3 months, p < 0.001). We could not detect any significant difference regarding the type of subsequent treatment, probably due to the small sample size for subgroup analysis.

### Toxicity profile

A total of 33 patients (94%) experienced grade 1 acute toxicities, and 17 patients (49%) developed grade 2 toxicities. The most common toxicities of grade ≥ 2 were skin reactions (n = 17), alopecia (n = 6), and nail loss (n = 2). Two (6%) SS patients experienced grade 3 toxicity (bacteremia) and were treated with antibiotics during the radiotherapy. There was no significant difference in radiation‐related toxicity grades regarding radiation dose, fractionation, and type of disease. We could not observe grade ≥ 3 treatment‐related toxicities with the subsequent therapies.

### Health‐related quality of life (HRQoL)

Regarding pretreatment itching, Skindex‐29, and EORTC QLQ‐C30 subscales, we could not detect any significant difference between eMF and SS patients. Based on the reported Skindex‐29 scores, a significant improvement in all subdomains was detected within 4 weeks after TSEBT initiation (Table 2). Among 28 patients (80%) with itching, more than two‐thirds (71%) had an improvement after TSEBT, and 25% of them had a complete response of pruritus.

**TABLE 1:** Patient, disease and treatment characteristics.

Characteristic	Erythrodermic MF, n = 14 (%)	Sézary syndrome, n = 21 (%)	*p*‐value
Median age, years (IQR)	64 (17)	69 (13)	0.18
Sex			
Male	11 (79)	16 (76)	1
Female	3 (21)	5 (24)	
Baseline performance status (ECOG)			
I	8 (57)	11 (52)	1
II	6 (43)	10 (48)	
Baseline mSWAT score, median (IQR)	91 (22)	100 (20)	0.78
Clinical symptoms at time of referral			1.0
Itching	14 (100)	21 (100)	
Hyperkeratosis	10 (71)	15 (71)	
Alopecia	10 (71)	14 (67)	
Itching scale before TSEBT (IQR)	8 (2)	8 (4)	0.49
LDH elevated	8 (57)	16 (76)	0.42
WBC elevated	3 (21)	9 (45)	0.28
Nodal involvement	9 (64)	17 (81)	0.42
Large‐cell transformation before radiation	3/12 (25)	2/15 (13)	0.63
Number of previous therapies (Range)	3 (1‐6)	4 (2‐8)	0.12
Median time from diagnosis to TSEBT, months (IQR)	35 (13)	28 (54)	0.96
Applied TSEBT dose regimens			1.0
8 Gy in 2 or 4 fractions	6 (43)	8 (38)	
12 Gy in 8 fractions	8 (57)	13 (62)	
Antibiotic therapy simultaneous to TSEBT	4 (29)	7 (33)	1.0
Second TSEBT course	2 (14)	5 (24)	0.67
Lymph nodes boost radiotherapy	6 (43)	13 (62)	0.32
Response to TSEBT			0.61
CR	1 (7)	1 (5)	
VGPR	8 (57)	7 (33)	
PR	4 (29)	10 (48)	
Maintenance or subsequent treatment in responders			0.69
Yes	9 (69)	14 (78)	
No	4 (31)	4 (22)	

*Abbr*.: CR, complete response; ECOG, Eastern Cooperative Oncology Group; IQR, interquartile range; LDH, lactate dehydrogenase; MF, mycosis fungoides; mSWAT, modified Severity Weighted Assessment Tool; PR, partial response; SS, Sézary syndrome; TSEBT, total skin electron beam therapy; VGPR, very good partial response; WBC, white blood cell count

**TABLE 2:** Skindex‐29, EORTC‐QLQ‐C30, itching, and modified severity‐weighted assessment tool scores in median (interquartile range) prior to and post radiation (n = 35).

	Skindex29 global score	Symptoms	Emotions	Functions	Question 18	EORTC QLQ‐C30 global score	Itching scale	mSWAT
Baseline	112 (37)	26 (9)	38 (14)	42 (21)	3.5 (1)	6 (5)	8 (2)	100 (20)
3 months after radiotherapy	79 (63)	18 (12)	25 (21)	29 (27)	2 (2)	7 (6)	2 (5)	14 (25)
Original p‐values^*^	0.0008	0.00002	0.00004	0.0004	0.002	0.007	0.000005	0.00003
Bonferroni‐corrected p‐values^**^	0.0064	0.00016	0.00032	0.0032	0.016	0.056	0.00004	0.00024

*Abbr*.: EORTC QLQ‐C30, European Organisation for Research and Treatment of Cancer Quality of Life Questionnaire‐Core 30; mSWAT, modified Severity Weighted Assessment Tool; Q18, item 18 of the EORTC QLQ‐C30 (pruritus intensity); Skindex‐29, 29‐item dermatology‐specific quality of life questionnaire

* Signed‐rank test

** Eight comparisons were performed

### Exploratory analysis of potential biomarkers

Given the prognostic potential of soluble biomarker testing in various hematologic malignancies, we leveraged a cohort of patients with eMF and SS who underwent serial laboratory and clinical assessments to explore candidate biomarkers. Our aim was to evaluate hematologic and serologic changes associated with clinical presentation and to correlate these with clinical outcomes.

A total of 33 patients (94%; 13 with eMF and 20 with SS) had paired blood samples collected at baseline and at first follow‐up (6–12 weeks after TSEBT). In comparison, 7 patients had paired samples collected at baseline and at disease progression. Initially, we compared baseline serum levels of potential biomarkers between patients with eMF and those with SS.

Regarding blood tumor burden, patients with SS showed elevated lymphocyte counts (median, 4185 vs. 1060 cells/µl; p = 0.02), CD4⁺ lymphocytes (median, 2170 vs. 575 cells/µl; p = 0.04), and a trend toward higher CD3⁺ counts (median, 1765 vs. 784 cells/µl; p = 0.065) compared to patients with eMF. There was no significant difference in the CD4:CD8 ratio (median, 4.5 vs. 3.2; p = 0.24). Baseline soluble PD‐L1 values were significantly correlated with skin disease burden (r = 0.608, p = 0.047). Interestingly, sIL‐2R values (median, 3279 vs. 833 pg/ml, p = 0.036) and LDH level (median, 308 vs. 234 pg/ml, p = 0.057) were noticeably elevated in SS cohort vs. eMF. All other biomarkers were similar in the eMF vs SS cohorts.

Following TSEBT, the leucocyte count reduced significantly after treatment (p < 0.001) in the whole cohort. In addition, SS patients had a significant decrease in median value of LDH (p = 0.005), Sézary cells (p = 0.003), CD3 (p = 0.006), CD4 (p = 0.003), CD19 (p = 0.05), sIL‐2R (p = 0.015), and sCD30 (p = 0.04) biomarkers between baseline and follow‐up, whereas those who had eMF had no significant change between baseline and follow‐up (p > 0.05). Conversely, in patients with eMF, there was a significant decrease in β_2_‐microglobulin (p = 0.03) and IgG levels (p = 0.05) from baseline to follow‐up. In contrast, LDH (p = 0.019) and soluble TARC levels (p = 0.043) significantly increased in cases of disease progression or relapse.

## DISCUSSION

In this study, we analyze the efficacy of a low‐dose TSEBT regimen in patients with eMF and SS and depict various exploratory objectives at baseline and follow‐up. The following findings emerged from our analysis: First, low‐dose TSEBT was associated with high ORR and rapid improvement of QoL scores. Second, the administration of subsequent treatment after low‐dose TSEBT in eMF/SS might be associated with clinical benefits. Third, we identified various potential biomarkers in the peripheral blood that warrants further investigation to explore any correlation with the best next systemic therapy.

Regarding quality of life data using Skindex‐29, other risk groups such as females, patients with elevated LDH, alopecia, and high mSWAT were associated with worse scores than other MF patients.^6^ Low‐dose TSEBT is usually associated with lower toxicities in advanced cases.^21^, ^22^ In addition, QoL proves to significantly improve within 4–8 weeks after radiation.^9^ Moreover, TSEBT is supposed to reduce tumor burden in the peripheral blood.^11^, ^12^ In a subgroup analysis of the SS cohort, patients showed a significant decrease in the median levels of LDH, white blood cell (WBC) count, Sézary cells, CD3⁺ T‐cell lymphocytes, CD4⁺ T‐cell lymphocytes, CD19⁺ B‐cell lymphocytes, soluble interleukin (IL)‐2 receptor (sIL‐2R), and sCD30⁺ T‐cell lymphocytes between baseline and follow‐up. Potential soluble biomarkers in the serum of patients before and after treatments are currently analyzed in a prospective studies.^15^, ^23^

Contemporary targeted therapies for MF and SS focus on various surface molecules expressed on tumor cells. Recently, mogamulizumab anti‐CCR4 antibody has been approved for advanced MF and SS patients.^24^, ^25^ According to the MAVORIC trial, mogamulizumab extended TTNT in patients with SS to up to 20 months in B2 disease, compared to 12 months in B1 and only 7 months in B0 disease. Meanwhile, the ORR was 46% in SS vs. 26% in B1, and 16% in B0 disease.^26^ However, the response rate in the blood compartment was higher than in the skin (68% vs. 42%), likely due to the presence of natural killer cells, which are essential for the antibody‐dependent cellular cytotoxicity (ADCC) mechanism of mogamulizumab.^26^ Possible combinations between mogamulizumab and TSEBT have been suggested and might be reasonable.^27^, ^28^, ^29^ In our analysis, the ORR for patients with eMF was 93%, with a TTNT of 42 months, compared to an ORR of 86% and a TTNT of 20 months in SS patients who received subsequent therapy after TSEBT. Natural killer cells did not decline after TSEBT, which might enable mogamulizumab efficacy as a subsequent treatment after TSEBT.^30^ Recently, Assaf et al. recommend the combination of mogamulizumab with radiotherapy from therapy initiation to enhance the outcomes.^31^

In a multicenter retrospective study, pegylated interferon alfa achieved a PR rate of 38% in patients with stage IIIA–IIIB MF (n = 8), with a median TTNT of 8 months.^32^ In stage IVA1 (n = 18), the ORR was comparatively higher at 56%, with a median TTNT of 11 months. While seven patients with stage IVA2 disease showed 57% ORR with 14 months TTNT. Two‐thirds of cases received combined therapy (most common with ECP or bexarotene) with superiority over monotherapy.^32^ The rate of grade ≥ 3 toxicities is ranging between 22% and 29%.^32^, ^33^ Similar to our data, the recently published EORTC consensus guidelines recommend interferons, oral retinoids, methotrexate therapy, and extracorporeal photopheresis as maintenance therapies for the treatment of MF/SS.^8^

Possible mechanisms of action of TSEBT in SS include the exposure of circulating malignant cells to low‐dose radiation as they pass through superficial dermal vessels. This exposure may activate the NF‐κB pathway, which in turn regulates relevant genes and cytokine expression.^34^ Moreover, radiotherapy induces various genic alterations in tumor cells involving the immune system, producing pro‐inflammatory signals, changing the tumor microenvironment, and recruiting tumor‐infiltrating lymphocytes (TILs).^35^ In addition, tumor cells recruit chemokines and tumor‐infiltrating lymphocytes (TILs) to the irradiated site, induce the production of reactive oxygen species (ROS) and kinase activation associated with DNA damage, and promote the presentation of natural killer cell‐activating ligands, thereby facilitating the recruitment of dendritic cells. These mechanisms suppress tumor cells in the local microenvironment and influence the growth of both irradiated and non‐irradiated lesions.^35^, ^36^ Therefore, additional immune modulatory treatments or immunotherapies might enhance the local and systemic antitumor immune responses following radiotherapy.^37^

Our study has several limitations, including the small sample size. However, given the rarity of the disease and the defined study period, we consider the findings to be relevant. Second, this is a complex and heterogeneous disease, and due to its rarity, conducting a study with a homogeneous patient population to generate meaningful clinical recommendations remains challenging. Additionally, in individual cases, the treating dermatologist decided on further treatment based on disease stage and comorbidities, which in some instances led to varying recommendations. Due to the COVID‐19 pandemic during the study period, paired blood samples for soluble biomarker analysis were available in only 86% of cases, and 40% of patients received ultra‐hypofractionated treatment regimens. In this study, more than half of the SS patients had secondary progression from MF, which is unusually high and likely attributable to the small and heterogeneous cohort. Despite these limitations, our findings support the feasibility of low‐dose TSEBT in combination with subsequent therapy in patients with eMF and SS and may serve as a foundation for future research.

It is important to note that disease control achieved with TSEBT is often transient, and the risk of relapse remains relatively high. Therefore, consideration of subsequent or maintenance therapy is recommended – particularly when supported by biomarkers indicating immune system activation and by the observed clinical benefits in patients receiving systemic treatment. In the future, it is important that additional prospective studies are conducted to increase the robustness of the findings. Accordingly, further randomized trials are needed to validate the efficacy of low‐dose TSEBT in combination with systemic therapies in patients with eMF and SS.

## CONCLUSIONS

In the present study, low‐dose TSEBT was shown to achieve disease control and may allow for repeated courses of radiotherapy in the future. Radiotherapy led to a marked improvement in patients' quality of life and was associated with relatively low toxicity. We recommend subsequent systemic therapy, as biomarker profiles suggest immune system activation and patients receiving systemic treatment may benefit from it.

## FUNDING SOURCES

This research has been supported by the Innovative Medical Research fund of the University of Munster Medical School (grant number: EL112102).

## CONFLICT OF INTEREST STATEMENT

K.E. received consulting and lecture fees from Kyowa Kirin and Gilead Sciences. J.P.N. received travel and congress participation funding from TEVA and Novartis, as well as consulting fees from TEVA, Almirall, Biogen, Novartis, Kyowa Kirin, Innate Pharma, Takeda, Actelion, UCB Pharma, and Recordati. S.M. received honoraria and travel funding from Kyowa Kirin. R.S. received honoraria from Kyowa Kirin, 4SCAg, Stemline, Recordati, and Innate Pharma. E.L. received honoraria from Takeda. C.P. received honoraria from MSD, BMS, Novartis, AbbVie, Sanofi, Merck, Serono, Sun Pharma, Pierre Fabre, LEO, and Allery Therapeutics.

The abstract was accepted for oral presentation and presented at the EORTC Cutaneous Lymphoma Tumour Group Annual Meeting 2024, held from 9 to 11 October 2024 in Lausanne, Switzerland. The abstract has been published in the European Journal of Cancer (EJC).

The remaining authors declare no conflict of interest.
